# Cell fibers promote proliferation of co-cultured cells on a dish

**DOI:** 10.1038/s41598-019-57213-0

**Published:** 2020-01-14

**Authors:** Ai Shima, Akane Itou, Shoji Takeuchi

**Affiliations:** 1 Department of Mechano-Informatics, Graduate School of Information Science and Technology, The University of Tokyo, Tokyo, Japan; 2 Institute of Industrial Science, The University of Tokyo, Tokyo, Japan; 3 International Research Center for Neurointelligence (WPI-IRCN), The University of Tokyo Institutes for Advanced Study (UTIAS), The University of Tokyo, Tokyo, Japan

**Keywords:** Tissue engineering, Cell growth

## Abstract

This paper describes a co-culture method using cell fiber technology. Cell fibers are cell-laden hydrogel microfibers, in which cells are cultured three-dimensionally and allowed to reach more mature state than the conventional two-dimensional cell culture. Cells in the cell fibers are encapsulated by alginate shell. Only cellular secretome is released into the surrounding environment through the shell while the cells were retained by the fiber. With their high handleability and retrievability, we propose to use the cell fibers for co-culture to ensure steady supply of cellular secretome. We cultured mouse C2C12 myoblasts with mouse 3T3 fibroblasts encapsulated in the cell fibers for two days. The number of C2C12 cells increased proportionally to the number of co-cultured 3T3 fibers, suggesting that the secretome of 3T3 fibers promoted survival and proliferation of C2C12 cells. We believe that cell fiber technology is a useful tool for co-culturing cells, and it will contribute to both basic cell biology and tissue engineering with its unique features.

## Introduction

Co-culture, in which two or more types of cells are cultured together, is a major method to study interactions between different types of cells *in vitro*. Cells interact with each other both directly (via physical contact) and indirectly (via secreted molecules; for example, cytokines, growth factors and hormones) and these interactions have an impact on cellular survival, proliferation, differentiation and maturation. To investigate the indirect cellular interactions, two major methods have been established; one using culture inserts and the other using conditioned medium. Culture inserts make upper and lower compartments in culture wells, which enables a concurrent co-culture. Two different types of cells are plated and cultured in the upper and lower compartments. Only cellular secretome, but not the cells themselves, is then transferred between those two compartments through the pores on the bottom of culture inserts, when the pore size is smaller than the cells. On the other hand, in the method that employs conditioned medium, a certain type of cells are cultured and the supernatant containing their secretome (conditioned medium) is collected to subsequently culture the other type of cells. These methods are often employed and have been shown to be effective to study various cellular interactions^1–3^. However, neither of them is highly space-efficient; the number of available cells that provides secretome is limited because of the culture area.

Cell fiber is a unique tool for culturing cells three-dimensionally for a long period until they differentiate into a mature tissue^4^. Cell fibers, which are cell-laden hydrogel microfibers formed by using a double-coaxial laminar-flow microfluidic device, consist of two parts; the core containing cells and extra cellular matrix (ECM) proteins such as collagen, and the alginate shell. Various types of cells have been shown to form three-dimensional (3D) tissues in cell fibers; for example, cardiomyocytes, vascular endothelial cells, nerve cells, smooth muscle cells and adipocytes^4–6^. Cell fibers enable a large number of cells to be packed together. The cells can easily access oxygen and nutrients in the culture medium, since thickness of the core containing the cells is kept a few hundreds of micrometers over the entire length. Also, they are mass-producible; by determining the flow rate and injection time of the core, cell fibers containing roughly the same number of cells can be repeatedly formed. Cell fibers are not only a useful tool for basic cell biology to study cellular behavior in a 3D culture, but also have the potential to be applied as grafts for cell therapy. It was demonstrated that the transplanted insulin-secreting cell fiber decreased blood glucose level in diabetic mice^4,7^. The advantage of cell fibers is that the alginate shell isolates the transplanted cells in the core from host immune system, which otherwise induces foreign-body reaction. The other advantage is their handleability (easily transplantable and retrievable).

**Figure 1:**
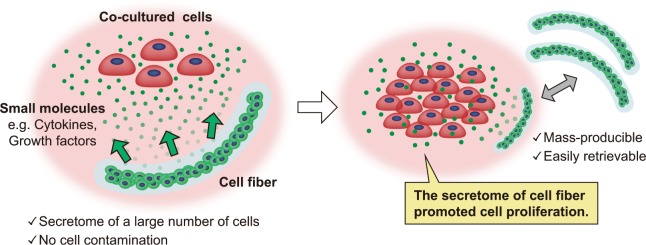
Conceptual illustration of this study. A large number of cells in the cell fiber secrete small molecules such as cytokines and growth factors into the culture medium through the hydrogel shell, while the cells are retained by the fiber. Since cell fibers are mass-producible and can be easily retrieved from the culture, they are a useful tool to supply cell-derived components to other cells in co-culture. This study investigated whether the secretome of cell fibers promoted proliferation of co-cultured cells.

In this study, we propose to use the cell fibers, which provide cell-derived components, for co-culture of different types of cells (the conceptual illustration of this study is shown in Fig. 1). Cell fibers contain a large number of cells in less volume and are mass-producible. They can also be easily retrieved after the co-culture. Here, we culture mouse 3T3 fibroblasts encapsulated in the cell fibers and mouse C2C12 myoblasts together to investigate whether the secretome of 3T3 fibers promotes the proliferation of C2C12 cells without causing cellular contamination.

## Results

### Co-culture of C2C12 cells and a 3T3 fiber without cellular contamination

**Figure 2:**
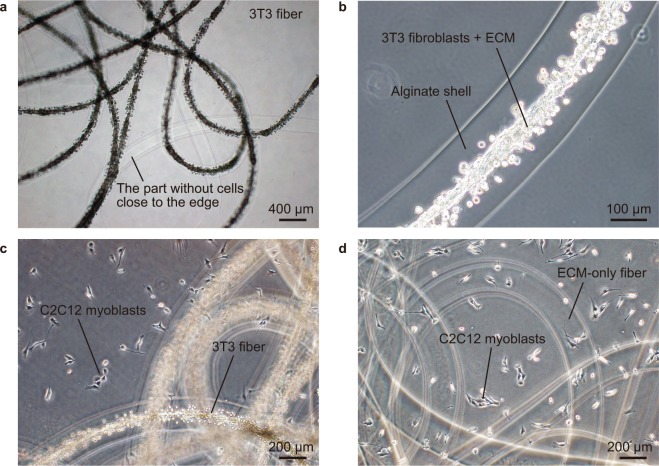
Co-culture of a 3T3 fiber and C2C12 cells. (**a**) Low-magnification and (**b**) high-magnification images of a 3T3 fiber three days after formation. The core consisting of 3T3 fibroblasts and ECM was sealed with the alginate shell. The part close to the edges did not contain the cells. (**c**) Co-culture of a 3T3 fiber and C2C12 cells. The day-3 3T3 fiber was transferred to C2C12 culture in a dish to initiate the co-culture. (**d**) Control culture of an ECM-only fiber and C2C12 cells.

First, we monitored the formation of 3T3 fibers and their handleability for co-culture. After three days of culture, 3T3 cells adhered to each other and formed fiber-based 3D tissues in the alginate shell of the cell fibers (Fig. 2a,b). The diameter of alginate shell of 3T3 fibers was about 220 μm, whereas the diameter of the core (cells and ECM) was about 70 μm on day 3. A certain length of both ends of the 3T3 fibers was composed of only alginate shell without the core containing cells (Fig. 2a), so that the cells would not leak out from the edges. Before starting co-culture with C2C12 cells plated on tissue culture dishes, the 3T3 fibers were observed carefully and confirmed to be intact. The entire length of cell fibers could be observed at low magnetic field by microscope. In case 3T3 fibers were found to have ruptures on the shell leading to leakage of the cells, they were not used for co-culture. The 3T3 fibers were cultured in Dulbecco’s modified Eagle medium (DMEM) containing 10% fetal bovine serum (FBS) after being formed. To avoid traces of FBS during transfer of the cell fibers into the serum-free C2C12 culture, the 3T3 fibers were washed in fresh DMEM (containing only antibiotics) for three times before being transferred. The cell fibers were then transferred to the dishes on which C2C12 cells were cultured to investigate the effect of cellular secretome of 3T3 fibers on C2C12 cell proliferation (Fig. 2c). No visible damage was observed after the cell fibers were washed and transferred by using pipets. During the co-culture for two days, 3T3 cells in the cell fibers did not increase substantially and no cellular leakage was observed in the C2C12 culture. These results suggested that 3T3 fibers were suitable for co-culture in terms of the handleability and sealing property.

### The effect of a 3T3 fiber on C2C12 cell proliferation

**Figure 3:**
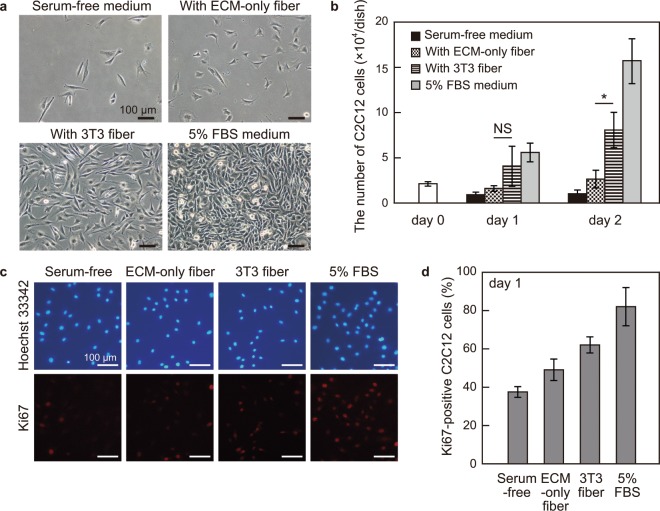
The effect of 3T3 fibers on C2C12 cell proliferation. (**a**) Images of C2C12 cells after being cultured for two days in serum-free medium, with an ECM-only fiber, with a 3T3 fiber and in medium containing 5% FBS. Scale bars; 100 μm. **(b**) The number of C2C12 cells per culture dish under each condition on day 0, day 1 and day 2. A 3T3 fiber promoted C2C12 cell proliferation significantly more (*P = 0.013; *t*-test) than an ECM-only fiber on day 2 did. Data are mean ± s.d. (n = 3). NS, not significant. (**c**) Images of immunofluorescence for the cell-proliferation marker, Ki-67 (red) under each condition on day 1. The cell nuclei were stained with Hoechst 33342 (blue). Scale bars; 100 μm. (**d**) The percentage of Ki67-positive C2C12 cells on day 1. The result showed a similar tendency in one of the cell number counting assays. Data are mean ± s.d. (n = 4).

We then investigated whether the cellular secretome of a 3T3 fiber could promote proliferation of C2C12 cells in the co-culture. As a control to check the effects of absorbed FBS in the ECM core and alginate shell, which was possibly left even after the wash, an ECM-only fiber was employed (Fig. 2d). Just before a 3T3 fiber was transferred (day 0), the number of C2C12 cells per dish was 2.1 × 10^4^ cells. C2C12 cells were then cultured with a 3T3 fiber or an ECM-only fiber in DMEM containing only antibiotics (serum-free medium), in the serum-free medium and in the C2C12 medium containing 5% FBS for two days (Fig. 3a). On day 2, the number of C2C12 cells cultured with a 3T3 fiber (8.1 × 10^4^ cells) was significantly higher (*P* = 0.013) than that cultured with an ECM-only fiber (2.7 × 10^4^ cells), whereas the difference between them was not statistically significant (*P* = 0.184) on day 1. The C2C12 cells cultured with an ECM-only fiber had hardly increased in two days. In the serum-free medium, the cell number even decreased, suggesting that the cells not only stopped proliferating but were also induced to undergo cell death. The co-culture with a 3T3 fiber promoted C2C12 cell proliferation, but the effect was not as high as the culture with 5% FBS (15.7 × 10^4^ cells on day 2) (Fig. 3b).

The expression of cell-proliferation marker Ki67^8^ was also analyzed under above-mentioned conditions by immunofluorescence (Fig. 3c). The percentage of Ki67-positive C2C12 cells was highest when cultured in the C2C12 medium containing 5% FBS, followed by the culture with a 3T3 fiber, the culture with an ECM-only fiber, and the culture in the serum-free medium, which was the similar pattern with the cell number (Fig. 3d).

We also cultured C2C12 cells in the conditioned medium (CM) obtained from confluent 3T3 cells plated on a 100-mm diameter dish (about 5 × 10^6^ cells) for comparison. To study the effect of cellular secretome on cell proliferation in the absence of serum, it was ensured that the CM did not contain FBS, unlike the conventional CM culture. On day 2, the number of C2C12 cells cultured in 3T3 CM was found to be reduced (1.5 × 10^4^ cells). The reason why CM was not as effective as a 3T3 fiber in promoting C2C12 cell proliferation might be the number of 3T3 cells cultured for CM (10^6^ order), which was much lower than that inside the cell fiber (10^7^ order). In addition, the lack of serum may have deteriorated the culture condition of 3T3 cells when cultured in a dish without ECM, which also led to insufficient amounts of the secretome.

### The effective factor in the secretome of a 3T3 fiber on C2C12 cell proliferation

Enzyme-linked immunosorbent assay (ELISA) detected fibroblast growth factor (FGF) 2 (13.8 ± 1.9 pg/ml) in the conditioned medium of a 3T3 fiber (serum-free medium cultured with a 3T3 fiber for two days without C2C12 cells), suggesting that one of the factors promoting C2C12 cell proliferation in the co-culture would be FGF2.

### The effect of cell fiber number on C2C12 cell proliferation

**Figure 4:**
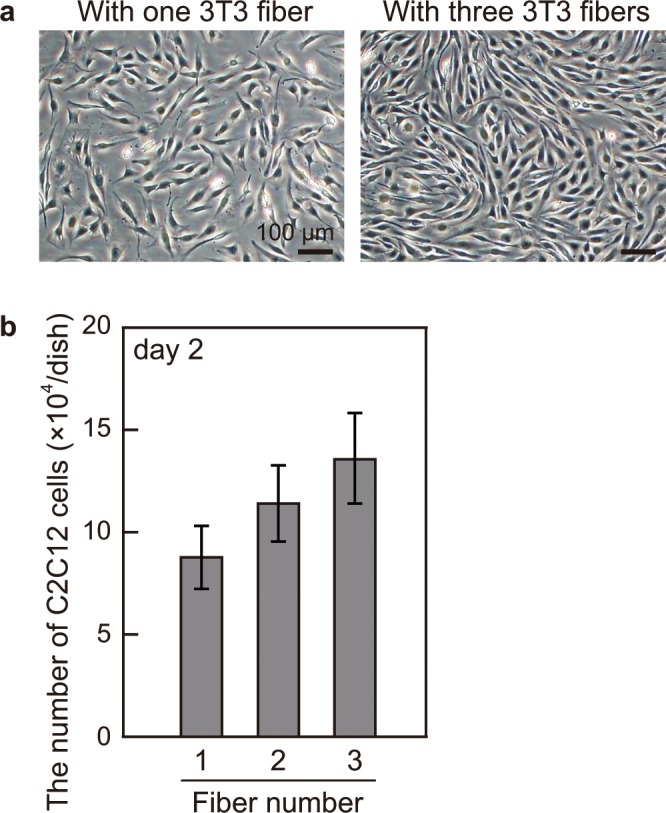
The effect of multiple 3T3 fibers on C2C12 cell proliferation. (**a**) Images of C2C12 cells after being cultured for two days with one or three 3T3 fibers. Scale bars; 100 μm. **(b**) The number of C2C12 cells per dish under each condition on day 2. The increase in the number of C2C12 cells was proportional to the number of 3T3 fibers. Data are mean ± s.d. (n = 4).

Finally, we investigated whether the effect of 3T3 fibers on C2C12 cell proliferation depended on the number of cell fibers. We cultured C2C12 cells with one, two and three 3T3 fibers for two days (Fig. 4a). The total volume of culture medium per dish was kept identical regardless of the number of 3T3 fibers. It was observed that C2C12 cells increased proportionally to the number of 3T3 fibers until day 2 (Fig. 4b). The number of C2C12 cells when cultured with three 3T3 fibers (13.5 × 10^4^ cells on day 2) was almost close to the number of C2C12 cells cultured with 5% FBS, suggesting that the effect of 3T3 fibers on C2C12 cell proliferation could match the effect of serum when multiple 3T3 fibers were co-cultured at the same time.

## Discussion

The current study proposed a co-culture system using cell fibers, micro-fiber-shaped cellular constructs in which cells were sealed by alginate gel. To demonstrate this concept, we co-cultured mouse C2C12 myoblasts plated on culture dishes with mouse 3T3 fibroblasts encapsulated in the cell fibers. The alginate shell was durable enough to seal 3T3 cells inside during the co-culture for two days. The 3T3 fibers did not show cell leakage during the co-culture. The number of C2C12 cells did not increase when cultured with an ECM-only fiber but quadrupled with a 3T3 fiber in two days even without FBS, which suggested that the cellular secretome of 3T3 fibers promoted C2C12 survival and proliferation. The cell proliferation-promoting effect was greater when the cells were co-cultured with multiple 3T3 fibers.

Cells secrete various types of cytokines and growth factors into the surrounding microenvironment and the secretome changes the phenotype of other cells via autocrine or paracrine signaling^9–11^. The cellular secretome has been used to culture other cells in co-culture system and shown to be effective. In the current study, it was also shown that co-culturing with 3T3 fibers promoted proliferation of C2C12 cells. It has been well-known for decades that fibroblasts have a paracrine effect on myoblasts^12^. The effective substances in the fibroblast secretome that stimulate myoblast proliferation were suggested to be growth factors such as FGF, insulin-like growth factor (IGF) and platelet-derived growth factor (PDGF)^12^. The current study also found that FGF2 was contained in the conditioned medium of a 3T3 fiber. The concentration of FGF2 was relatively low in the whole conditioned medium, but it would be higher in the immediate vicinity of 3T3 fibers in the co-culture. Recently, cellular secretome such as one from mesenchymal stem cells^13–15^ and myokine^16–18^ have been attracting attention in terms of their potential therapeutic effects, though some of them yet to be identified. It is difficult to determine the chemical composition of the secretome. However, we believe that cellular secretome is useful to promote cell growth in co-culture as some of the cell-derived components have not been artificially synthesized yet.

According to our results, the effect of 3T3 fibers on C2C12 cell proliferation was much greater than that of conditioned medium (CM) from 3T3 cells. We assume that the main reason for this observation was the concentration of effective chemical factors in CM, which was about ten times lower than that of factors secreted in the co-culture with a 3T3 fiber, since one tenth of 3T3 cells encapsulated in a 3T3 fiber were used to obtain the CM. In addition, 3T3 fibers could supply the secretome continuously and concurrently to C2C12 cells. C2C12 cells also could interact and change the behavior of 3T3 cells in the cell fibers, though no obvious effects on 3T3 fibers were observed in the current study probably because of the small number of plated C2C12 cells. Note that CM used in this study did not contain FBS unlike the conventional CM culture, in which the cell-derived components are additionally incorporated into the general culture medium containing animal serum.

Animal serum such as FBS is essential for cell survival and proliferation *in vitro*. However, serum has its own drawbacks; for example, rising price, ethical concerns, possible pathogenicity, lot-to-lot variation and undefined animal-derived components^19^. We demonstrated that the cell-derived components from a commonly used cell line, 3T3, could become a substitute for serum when packed at sufficiently high cell concentration in a cell fiber and multiple cell fibers were applied. Cell lines are commercially available at a relatively lower price and pose no ethical issues. Due to their high proliferation ability, cell lines can be less expensive alternatives to animal serum when used in combination with the cell fiber technology. Not only general cell lines but also genetically engineered cells (overexpressing cells) or antibody-producing cells (hybridomas) can be encapsulated in cell fibers to oversecrete the intended chemical factors or to produce monoclonal antibodies, respectively.

In conclusion, we demonstrated that 3T3 fibers promoted proliferation of co-cultured C2C12 cells. The cell fiber technology was useful to provide cellular secretome to the co-cultured cells. It is important in the research field of basic cell biology to elucidate various cellular interactions through paracrine signaling and study how the cells function in harmony in living organisms. We believe that the cell fiber technology will contribute to studies on cellular interactions with its unique features; scalability and handleability. Co-culture using cell fibers offers another advantage to the field of tissue engineering. Cell fibers can be co-cultured with the target cells not only in the standardized culture dishes but also in large tanks for industrial scale cell culture or original 3D culture devices, which have become more and more popular in recent years in the field of tissue engineering. Co-culture can be continued for more than two days by exchanging cell fibers regularly. Cell fibers can be retrieved after co-culture and the cultured tissues can be obtained without cellular contamination. We showed that the cell fibers can effectively provide secretome and can function as a less expensive alternative to animal serum. The compatibility of cell fibers with mass cell culture facilitate their contribution to the industrial field, for example cost-effective development of cultured meat^19,20^ in the future.

## Methods

### Cell culture

Mouse C2C12 myoblasts^21^ were plated on tissue culture dishes coated with 1% gelatin (Sigma) at 1 × 10^4^ cells per 35-mm diameter dish and cultured in DMEM (4500 mg/mL glucose) (Merck) containing 5% FBS (MP Biomedicals) and Penicillin-Streptomycin (Merck) (C2C12 medium) for 24 h before initiating co-culture with cell fibers or the control culture. For the cell fiber formation, mouse 3T3 fibroblasts^22^ were expanded in DMEM (4500 mg/mL glucose) containing 10% FBS and Penicillin-Streptomycin (3T3 medium). After the formation of 3T3 fibers and ECM-only fibers (mentioned below), both types of fibers were incubated in the 3T3 medium for three days. For the co-culture of C2C12 cells and 3T3 fibers, the 3T3 fibers were confirmed to be intact (no leakage of cells from the core) by microscopic observation and washed with DMEM (without serum) for three times to get rid of FBS on the surface. One, two or three 3T3 fibers were then added into the culture of C2C12 cells in 35-mm diameter dishes with 2 mL of DMEM containing only antibiotics (serum-free medium). As a negative control, the ECM-only fibers were washed and cultured with C2C12 cells. For other controls, the C2C12 medium (containing 5% FBS) and the serum-free medium were tested. For isolating conditioned medium (CM) from 3T3 cells, 3T3 cells were cultured in the 3T3 medium until they became confluent in 100-mm diameter dishes. The cells were then washed and incubated in the serum-free medium for 24 h and the supernatant was collected. The supernatant was centrifuged and filtered through a polyvinylidene difluoride (PVDF) membrane filter (0.22 μm pore) to get rid of the cells and used as a CM to culture C2C12 cells. All cultures were maintained in 5% CO_2_ at 37 °C. The cell images were acquired with IX71 inverted research microscope (Olympus Life Science).

### Cell fiber formation

The cell fibers were formed by using the double-coaxial laminar-flow microfluidic device assembled from pulled glass capillary tubes, rectangular glass tubes and connectors as previously reported^4^. To form the core-shell 3T3 fibers, three types of solutions were prepared: (1) 3T3 cells-containing pre-gel solution of a mixture of two types of collagen, AteloCell IAC-50 (Koken, Japan) and Cellmatrix Type I-A (Nitta Gelatin, Japan) as ECM for the core; (2) pre-gel solution of 1.5% Na-alginate (Fujifilm Wako Pure Chemical, Japan) for the shell; (3) 100 mM CaCl_2_, 3% glucose solution for the sheath stream. Each 3T3 fiber was formed to contain 4 × 10^7^ cells per fiber on the calculation with the certain length of both ends lacking cells (only alginate shell) to completely seal the cells. For the control fibers, only ECM without the cells was used to make the core (ECM-only fiber). The width of the shell and core parts of 3T3 fibers in randomly selected 18 fields from four different cell fiber formation trials were measured by using the imaging software cellSens (Olympus Life Science).

### Cell counting

C2C12 cells were trypsinized after washing twice with PBS (the 3T3 fiber was taken out in such cases) and re-suspended in 1 mL of the C2C12 medium, just before initiating co-culture with a 3T3 fiber (day 0) and after being co-cultured for one or two days (day 1 and day 2, respectively). The cell number per dish was counted three times by using cell-counting plate (WakenBtech, Japan) and averaged. Under each experimental condition, three or four culture dishes from different cell culture trials were assayed. The number of cells per dish was shown as the mean ± standard deviation (s.d.). The significance between the cell number with a 3T3 fiber and that with an ECM-only fiber was evaluated by *t*-test.

### Immunofluorescence for Ki-67

C2C12 cells were fixed with 4% paraformaldehyde/PBS after one-day co-culture with a 3T3 fiber, when the cells under all experimental conditions were supposed to be in proliferating phase. The cells were permeabilized with 0.2% triton X-100/PBS, incubated with 1% BSA/PBS for blocking and treated with the primary antibody, rabbit polyclonal anti-Ki67 antibody (Abcam). Alexa Fluor 594 goat anti-rabbit IgG (Thermo Fisher Scientific) was used as the secondary antibody. Cell nuclei were stained with Hoechst 33342 (Thermo Fisher Scientific). The immunofluorescence images were taken with IX71 inverted research microscope. The percentages of Ki67-positive cells to the total cells were calculated by counting more than 300 cells in randomly selected six fields per dish in each condition. Four culture dishes from different cell culture trials were assayed and the results were shown as the mean ± s.d.

### ELISA for the detection of the secretome of a 3T3 fiber

Two mL each of DMEM containing only antibiotics (serum-free medium) cultured with a 3T3 fiber for two days without C2C12 cells (conditioned medium of a 3T3 fiber) from three culture dishes was analyzed. The conditioned medium was centrifuged at 400 g for 10 min at 4 °C and the supernatant was collected and stored at −80 °C. ELISA was performed using Mouse/Rat FGF basic/FGF2 Quantikine ELISA Kit (R&D Systems) according to the manufacturer’s instructions. The signal was detected by using Cytation 5 Cell Imaging Multi-Mode Reader (BioTek Instruments). The concentration of FGF2 in the conditioned medium of a 3T3 fiber was evaluated by a calibration curve and shown as the mean ± s.d.

## Data Availability

The data that support the findings in this study are available from the corresponding author upon reasonable request.

## References

[1] Seo K, Suzuki T, Kobayashi K, Nishimura T (2019). Adipocytes suppress differentiation of muscle cells in a co-culture system. Anim. Sci. J..

[2] Ostrovidov S (2017). Three-dimensional co-culture of C2C12/PC12 cells improves skeletal muscle tissue formation and function. J. Tissue Eng. Regen. Med..

[3] Shima A (2011). IGF-I and vitamin C promote myogenic differentiation of mouse and human skeletal muscle cells at low temperatures. Exp. Cell Res..

[4] Onoe H (2013). Metre-long cell-laden microfibres exhibit tissue morphologies and functions. Nat. Mater..

[5] Hsiao AY (2015). Smooth muscle-like tissue constructs with circumferentially oriented cells formed by the cell fiber technology. PLoS One..

[6] Hsiao AY, Okitsu T, Teramae H, Takeuchi S (2016). 3D tissue formation of unilocular adipocytes in hydrogel microfibers. Adv. Healthc. Mater..

[7] Ozawa F, Okitsu T, Takeuchi S (2017). Improvement in the mechanical properties of cell-laden hydrogel microfibers using interpenetrating polymer networks. ACS Biomater. Sci. Eng..

[8] Sellathurai J, Cheedipudi S, Dhawan J, Schrøder HD (2013). A novel *in vitro* model for studying quiescence and activation of primary isolated human myoblasts. PLoS One..

[9] Skalnikova H, Motlik J, Gadher SJ, Kovarova H (2011). Mapping of the secretome of primary isolates of mammalian cells, stem cells and derived cell lines. Proteomics..

[10] Nicholson T, Church C, Baker DJ, Jones SW (2018). The role of adipokines in skeletal muscle inflammation and insulin sensitivity. J. Inflamm..

[11] Bomb R (2016). Myofibroblast secretome and its auto-/paracrine signaling. Expert. Rev. Cardiovasc. Ther..

[12] Quinn LS, Ong LD, Roeder RA (1990). Paracrine control of myoblast proliferation and differentiation by fibroblasts. Dev. Biol..

[13] Praveen Kumar L (2019). The mesenchymal stem cell secretome: a new paradigm towards cell-free therapeutic mode in regenerative medicine. Cytokine Growth Factor Rev..

[14] Dietrich J (2019). Analysis of lacrimal gland derived mesenchymal stem cell secretome and its impact on epithelial cell survival. Stem Cell Res..

[15] Harrell CR (2019). Molecular mechanisms responsible for therapeutic potential of mesenchymal stem cell-derived secretome. Cells..

[16] Whitham M, Febbraio MA (2016). The ever-expanding myokinome: discovery challenges and therapeutic implications. Nat. Rev. Drug Discov..

[17] Son JS (2017). Effects of exercise-induced apelin levels on skeletal muscle and their capillarization in type 2 diabetic rats. Muscle Nerve..

[18] Otaka N (2018). Myonectin is an exercise-induced myokine that protects the heart from ischemia-reperfusion injury. Circ. Res..

[19] Bhat ZF, Fayaz H (2011). Prospectus of cultured meat—advancing meat alternatives. J. Food Sci. Technol..

[20] Post MJ (2012). Cultured meat from stem cells: challenges and prospects. Meat Sci..

[21] Yaffe D, Saxel O (1977). Serial passaging and differentiation of myogenic cells isolated from dystrophic mouse muscle. Nature..

[22] Todaro GJ, Green H (1963). Quantitative studies of the growth of mouse embryo cells in culture and their development into established lines. J. Cell Biol..

